# An innovative cell-laden α-TCP/collagen scaffold fabricated using a two-step printing process for potential application in regenerating hard tissues

**DOI:** 10.1038/s41598-017-03455-9

**Published:** 2017-06-09

**Authors:** Won Jin Kim, Hui-Suk Yun, Geun Hyung Kim

**Affiliations:** 1 Department of Biomechatronic Engineering, College of Biotechnology and Bioengineering, Sungkyunkwan University (SKKU), Suwon, South Korea; 2 Powder and Ceramics Division, Korea Institute of Materials Science (KIMS), Changwon, South Korea

## Abstract

Cell-laden scaffolds are widely investigated in tissue engineering because they can provide homogenous cell distribution after long culture periods, and deposit multiple types of cells into a designed region. However, producing a bioceramic 3D cell-laden scaffold is difficult because of the low processability of cell-loaded bioceramics. Therefore, designing a 3D bioceramic cell-laden scaffold is important for ceramic-based tissue regeneration. Here, we propose a new strategy to fabricate an alpha-tricalcium-phosphate (α-TCP)/collagen cell-laden scaffold, using preosteoblasts (MC3T3-E1), in which the volume fraction of the ceramic exceeded 70% and was fabricated using a two-step printing process. To fabricate a multi-layered cell-laden scaffold, we manipulated processing parameters, such as the diameter of the printing nozzle, pneumatic pressure, and volume fraction of α-TCP, to attain a stable processing region. A cell-laden pure collagen scaffold and an α-TCP/collagen scaffold loaded with cells via a simple dipping method were used as controls. Their pore geometry was similar to that of the experimental scaffold. Physical properties and bioactivities showed that the designed scaffold demonstrated significantly higher cellular activities, including metabolic activity and mineralization, compared with those of the controls. Our results indicate that the proposed cell-laden ceramic scaffold can potentially be used for bone regeneration.

## Introduction

Biomedical scaffolds have improved with the development of tissue engineering technology. The scaffolds are widely applied for regenerating tissues and organs such as skin, nerves, bladder, bone, and blood vessels^1–3^. However, ideal biomedical scaffolds, for the successful regeneration of tissues, are still lacking. The appropriate scaffolds need to have suitable mechanical properties to enable the structural integrity of the implant in the body under the conditions of biophysical and biochemical stress; these properties must include a highly porous structure, good biocompatibility, and biodegradability without the build-up of toxic by-products^4^.

Bioprinting, which is controlled with a computer-aided design system, is used in tissue engineering applications because it can fabricate various complex microscale structures in a layer-by-layer manner. A 3D printing method, called cell printing, uses cell-laden bioink and can overcome the shortcomings of conventional 3D scaffolds printed without cells. One such shortcoming is non-homogeneous cell seeding/growth within cell-seeded scaffolds. Cell printing can directly print any cells, using a cell-laden hydrogel (bioink), on the required region of the scaffold, resulting in successful 3D tissue architecture with homogeneous cell proliferation and even differentiation. Various methods, such as dispensing with a micro-sized nozzle using pneumatic/mechanical pressure, ink-jet printing with heat, acoustic waves, piezoelectric transducers (PZT), and laser printing, have been used to fabricate cell-laden structures^5–10^. Using the crosslinking properties of bioinks has improved the process of cell printing. However, residual issues, such as poor mechanical properties and printability of cell-laden, hydrogel-based bioinks, make it difficult to obtain the highly porous, pore-interconnected structure and realistic macro-scale scaffolds^7–11^. The mechanical properties of cell-laden scaffolds for the regeneration of bone tissue are particularly important because they can directly affect cell-morphology and osteogenic differentiation^12, 13^.

Cell-laden scaffolds, supplemented with synthetic polymers such as poly(ε-caprolactone) (PCL) and polylactic acid^14, 15^, as well as bioceramics manufactured from tricalcium phosphate (TCP) and hydroxyapatite (HA)^16, 17^, have been used to overcome these issues. Yun *et al*. designed a core/shell cell-laden structure fabricated by a 3D printing system^16^. The core region of this structure, α-TCP, was printed using a screw-rotating device; simultaneously, an alginate, laden with MC3T3-E1 cells, was printed on the shell region using pneumatic pressure. Using the α-TCP region in the core endowed the cell-laden scaffold with considerably enhanced mechanical strength and stable *in situ* cell viability during a prolonged culture period.

Here, we introduce a new bioceramic-based cell-printing technique and a cell-laden ceramic structure that shows enhanced mechanical properties and sufficiently high cellular activity. To produce the cell-laden scaffold, we employed α-TCP and type I collagen as a cell-delivering hydrogel, because bone tissue consists of calcium phosphate-based inorganic components and collagen-based organic components. TCP is widely used in bone regeneration because of its outstanding bioactivity and osteoconductivity^18^. TCP consists of two main crystal structures, α-TCP and β-TCP. α-TCP is more soluble compared with β-TCP and α-TCP can have a cementic reaction, which hardens the bioceramic to form a calcium-deficient hydroxyl apatite (CDHA) in an aqueous condition, such as a culturing condition in minimum essential media alpha (α-MEM)^16^. therefore, when used for bone tissue regeneration, α-TCP shows a more rapid bone formation *in vivo* relative to that of β-TCP, although the two TCPs have a similar chemical structure^19, 20^. Using α-TCP and collagen, we manufactured a ceramic-based cell-laden scaffold in two steps. First, to obtain a mechanically stable structure, we printed a porous layer consisting of micro-sized α-TCP/collagen struts without cells; then, a cell-laden collagen bioink was printed onto the α-TCP/collagen struts. The two-step process was repeated several times to form a 3D porous cell-laden ceramic scaffold (cell-laden α-TCP/collagen structure fabricated with a cell-printing process, TC-CPRINT). The various physical and biological activities of the preosteoblasts (MC3T3-E1), in the cell-laden ceramic scaffold, were analyzed. We compared the results with two controls: (1) a cell-laden collagen scaffold (CLCS), which was fabricated with cell-laden collagen cross-linked with tannic acid (TA) solution, and (2) an α-TCP/collagen scaffold, which was dipped into cell-laden collagen solution (cell-laden α-TCP/collagen structure fabricated with a cell-dipping process, TC-CDIP). After culturing the cells on the scaffolds, we compared the homogeneity and metabolic activities of the grown cells. We also tested the osteogenic activities of the scaffolds, including those of alkaline phosphatase (ALP) and osteopontin (OPN), as well as calcium deposition.

## Results and Discussion

### Fabrication of cell-loaded structures

**Figure 1:**
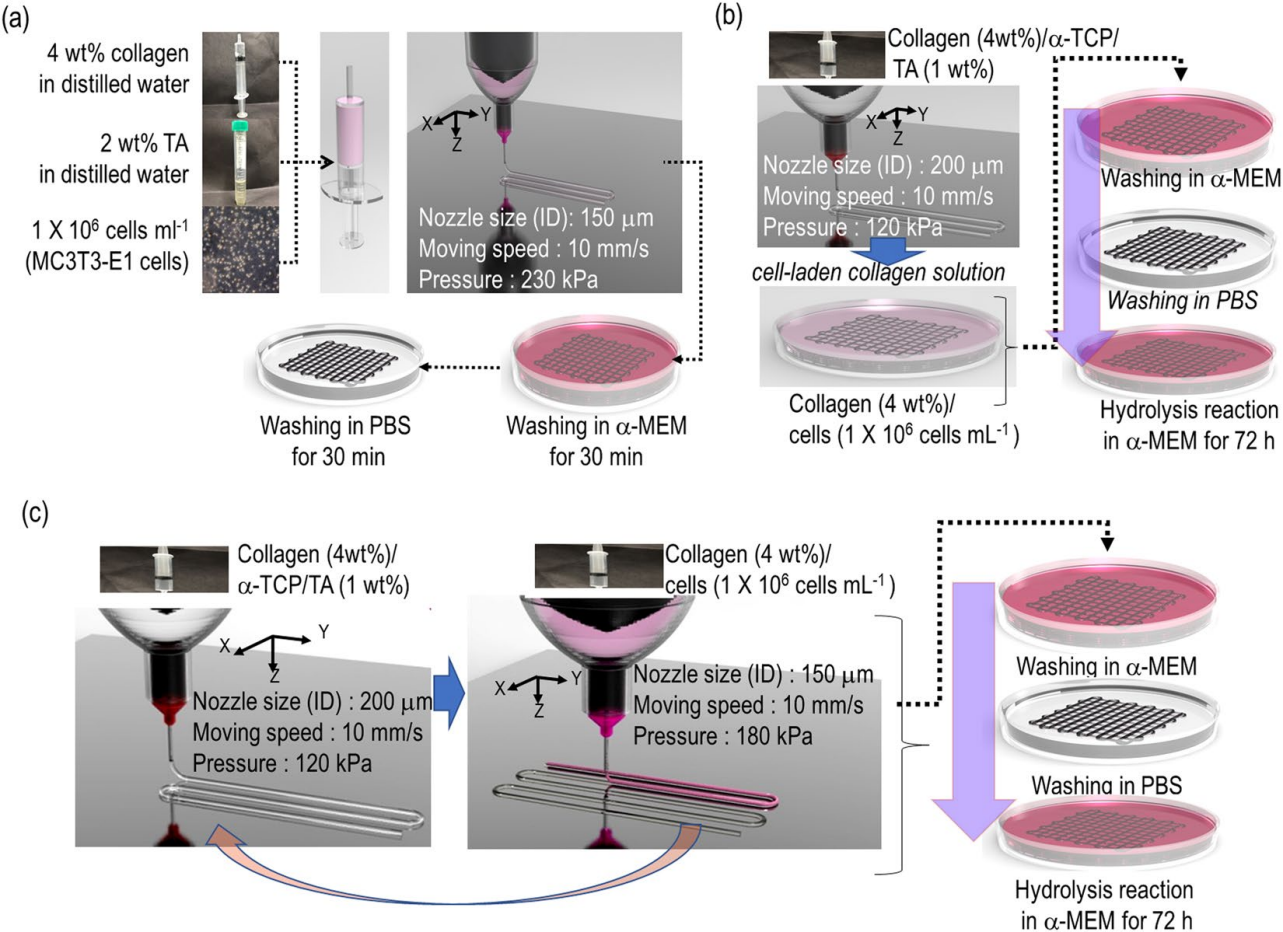
Fabrication schematics of cell-loaded scaffolds. (**a**) A cell-laden collagen scaffold, (**b**) a cell-laden α-TCP/collagen scaffold loaded using a dipping method, and (**c**) a 3D cell-laden α-TCP/collagen scaffold loaded using cell printing. The schematic diagram was drawn by W.J. Kim.

As a control, we fabricated a multi-layered, porous, cell-laden collagen scaffold (CLCS) using 4 wt% collagen mixed with MC3T3-E1 cells (1 × 10^6^ cells mL^−1^) and 2 wt% tannic acid (Fig. 1a).

The fabrication schematics for the two cell-loaded α-TCP/collagen scaffolds (TC-CDIP and TC-CPRINT) are described in Fig. 1b and c, respectively. To fabricate TC-CDIP, a 3D α-TCP/collagen mesh structure was dipped into a cell-laden collagen solution (4 wt% collagen and MC3T3-E1 cells at the density of 1 × 10^6^ cells mL^−1^) (Fig. 1b).

The α-TCP/collagen struts were printed as shown in Fig. 1c. Then, the cell-laden collagen solution was printed onto the struts. These steps were repeated several times to achieve the cell-laden 3D mesh structure (TC-CPRINT). Finally, the two cell-loaded scaffolds (TC-CDIP and TC-CPRINT) were rinsed in α-MEM medium and PBS.

### The effect of the crosslinking agent, tannic acid, on the fabrication of α-TCP/collagen structure

**Figure 2:**
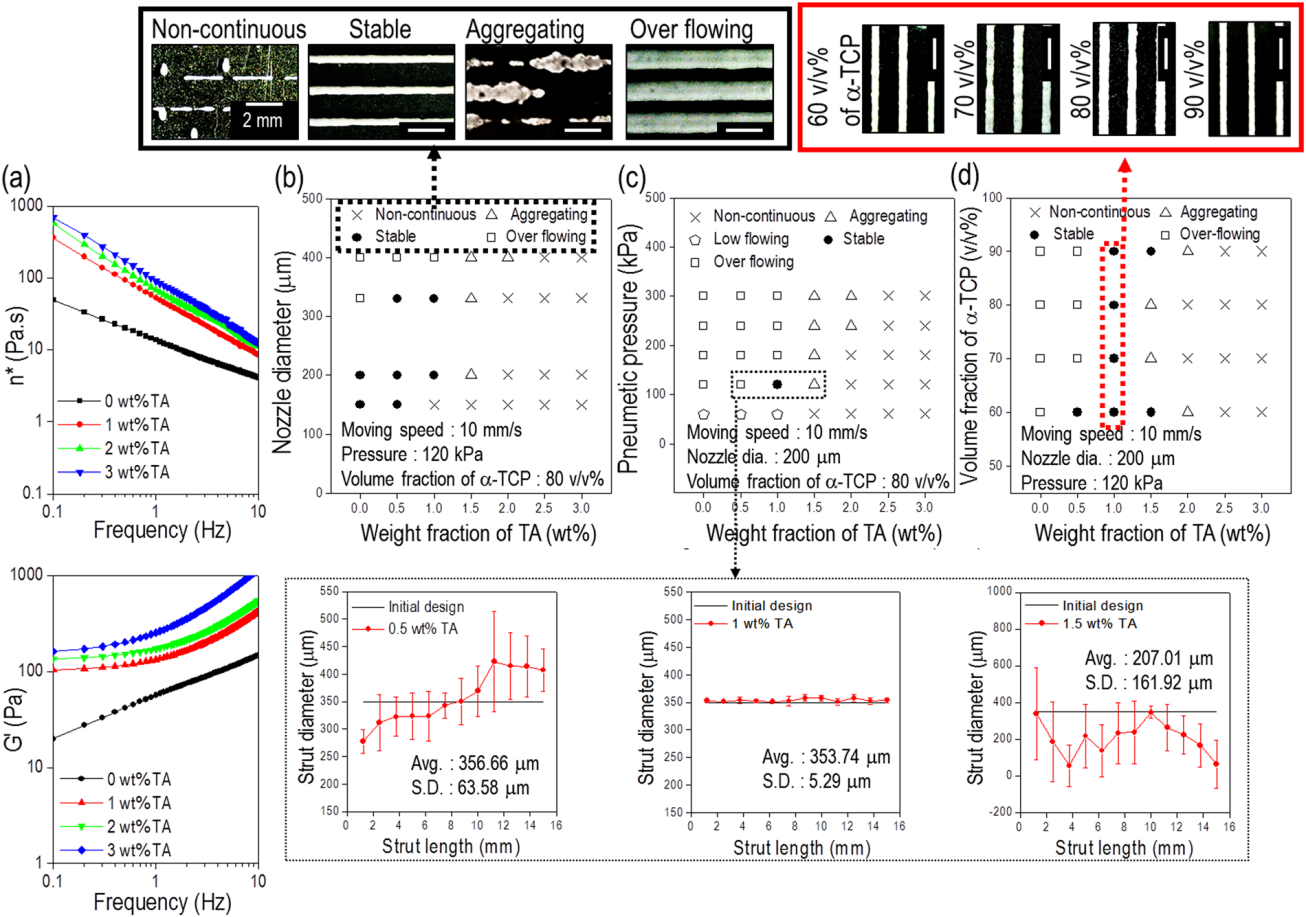
Rheological properties and processing diagrams. (**a**) Rheological properties (n*, complex viscosity and G’, storage modulus) of cell-laden collagen solution crosslinked with various weight fractions of tannic acid (TA). Processing diagrams demonstrating stable formation of α-TCP/collagen struts for processing parameters, (**b**) printing nozzle diameter, (**c**) pneumatic pressure of printing process, and (**d**) volume fraction of α-TCP, for the various weight fractions of TA.

Figure 1b shows a schematic describing the fabrication procedure of the α-TCP/collagen structure. To achieve a stable mesh structure consisting of a ceramic and collagen type-I, we used tannic acid (TA) as a crosslinking agent for collagen. Tannic acid crosslinks collagen by configuring numerous hydrogen bonds between collagen and TA^23^. To determine how various weight fractions of TA affect the crosslinking of collagen, we measured the storage modulus (G’) and complex viscosity (n*) of the collagen/TA mixtures. Figure 2a shows the rheological properties of the various mixtures of collagen and TA for the frequency sweep at 1% strain and 30 °C. The results show that the rheological properties (G’ and n*) were gradually increased as the concentration of TA increased in the mixture.

To determine the optimal range for processing parameters (weight fraction of TA, nozzle size of the dispensing system, pneumatic pressure, and volume fraction of α-TCP) for the stable fabrication of a porous mesh structure, we manipulated the parameters using a single line test. Figure 2b–d shows the stable/unstable processing range with respect to nozzle size, pneumatic pressure, and volume fraction of α-TCP, respectively, at various TA concentrations. As the nozzle size and weight fraction of TA increased, we observed unstable flow or no flow; this was because of non-uniform/agglomerated flow, or high viscosity, of the mixture, caused by a high degree of collagen crosslinking (Fig. 2b). As shown in Fig. 2c, an overly high pneumatic pressure (120 kPa) caused a high flow rate of the mixture; therefore, the designed strut diameter was not achieved. For the pneumatic pressure of 120 kPa, an appropriate weight fraction of TA would obtain the stable strut diameter of the printed structure, as shown in the magnified box of Fig. 2c. A low degree of collagen crosslinking (0.5 wt% of TA) also causes instability in a larger strut diameter (356.7 ± 63.5 μm), while a high degree of crosslinking (1.5 wt% of TA) can cause instability in a smaller diameter (207.0 ± 161.9 μm) of the printed struts. However, the volume fraction of α-TCP, at the same TA concentration (1 wt%), does not significantly affect the size stability of the strut (Fig. 2d). Based on the analysis using single strut printing, we selected the appropriate settings for the parameters: nozzle size of 200 μm, pneumatic pressure of 120 kPa, and 1 wt% for the concentration of TA.

**Figure 3:**
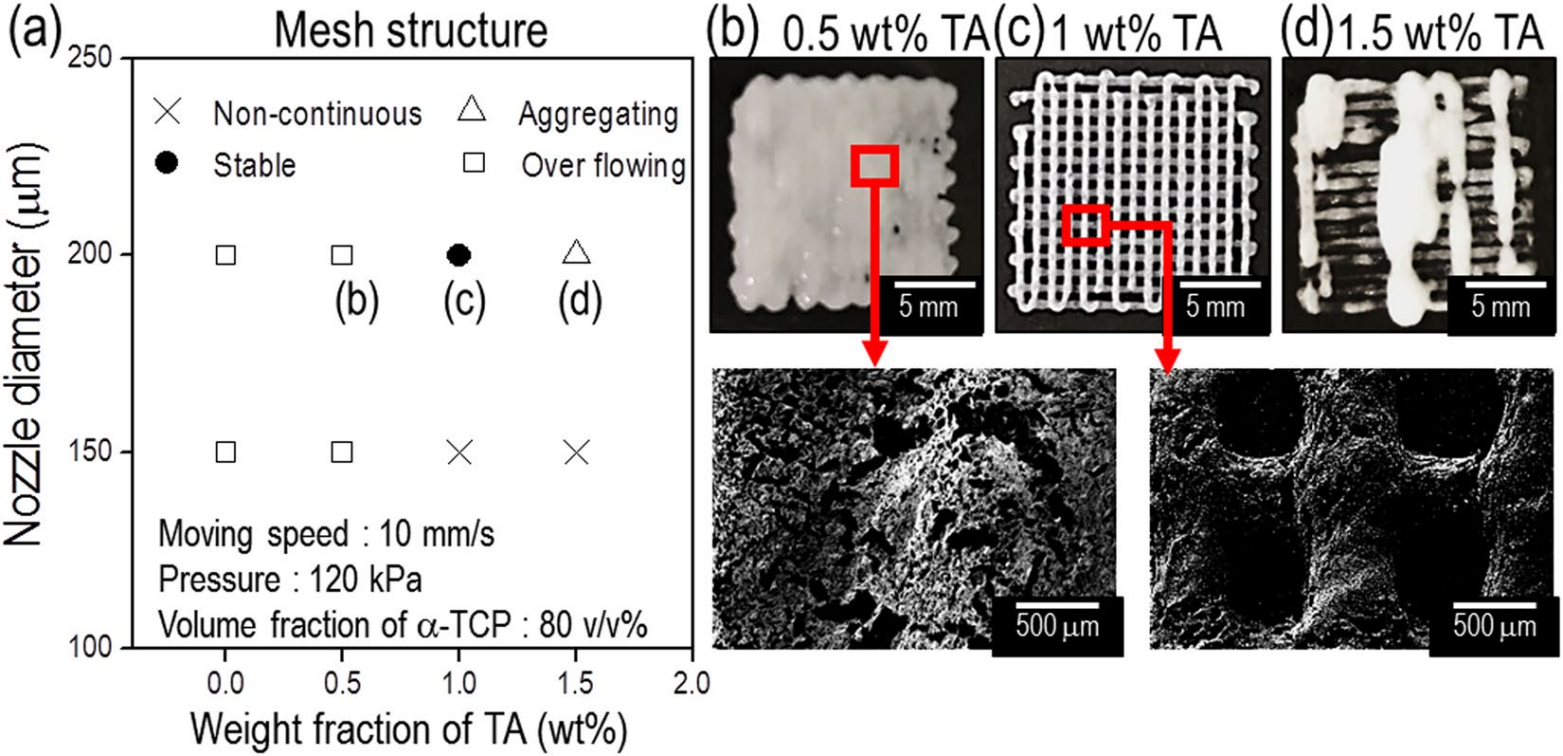
A processing diagram for a mesh structure. (**a**) A processing diagram for obtaining a 3D mesh structure, consisting of α-TCP/collagen struts, for a nozzle diameter of the 3D printer and various concentrations of tannic acid. Optical and SEM images of the fabricated mesh structure for the various concentrations (**b**) 0.5 wt%, (**c**) 1 wt%, and (**d**) 1.5 wt% of tannic acid at a constant nozzle diameter of 200 μm.

Using the selected pneumatic pressure (120 kPa) and volume fraction (80 v/v%) of α-TCP, we assessed the fabricating stability for obtaining a multi-layered mesh structure at various TA concentrations (Fig. 3a). Among the tested concentrations of TA, 1 wt% TA, and a nozzle diameter of 200 μm, resulted in the stable formation of a multi-layered α-TCP/collagen mesh structure (Fig. 3b-d).

**Figure 4:**
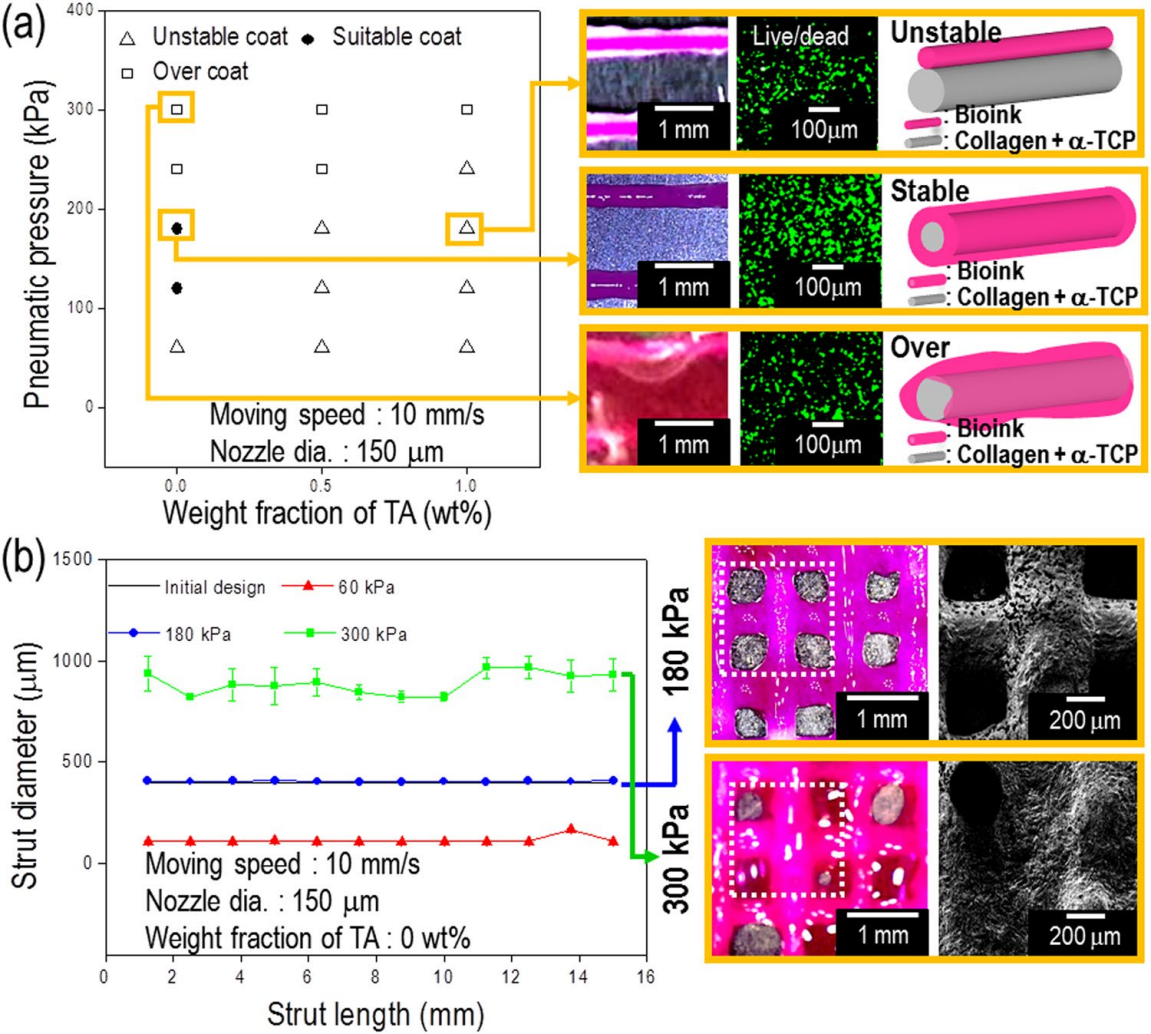
A processing diagram for cell-printing process. (**a**) Pneumatic pressure and tannic acid in the cell/collagen solution affect the coating of the α-TCP/collagen struts. Optical images show the stable and unstable statuses of the coated cell-laden collagen, and *in situ* live (green)/dead (red) staining, after printing with the cell-laden collagen. (**b**) Strut diameter, printed with the cell-laden collagen solution, for various pneumatic pressures. Optical and scanning electron microscopy (SEM) images for cell-laden collagen, printed on the α-TCP/collagen mesh structure, for the pneumatic pressures of 180 kPa and 300 kPa.

After fabricating the α-TCP/collagen mesh structure, we printed the cell-laden collagen bioink onto the struts. Figure 4a shows that the TA weight fraction and pneumatic pressure affect the printability of cell-laden collagen onto α-TCP/collagen struts at a fixed nozzle diameter (150 μm).

Non-crosslinked collagen bioink showed the most stable deposition onto the strut, as shown in the schematic. As shown in the results of the live (green)/dead (red) assay, the printed cells were unaffected (cell viability = 94%) during the printing process. However, when using non-crosslinked collagen bioink, an overly high pneumatic pressure can cause an over-flow on the struts, as shown in Fig. 4b. We obtained optimal conditions for the printing of cell-laden collagen solution using the nozzle size of 150 μm, pneumatic pressure of 180 kPa, and non-crosslinked collagen bioink.

### Pore geometry of cell-laden collagen and cell-laden α-TCP/collagen scaffolds

**Figure 5:**
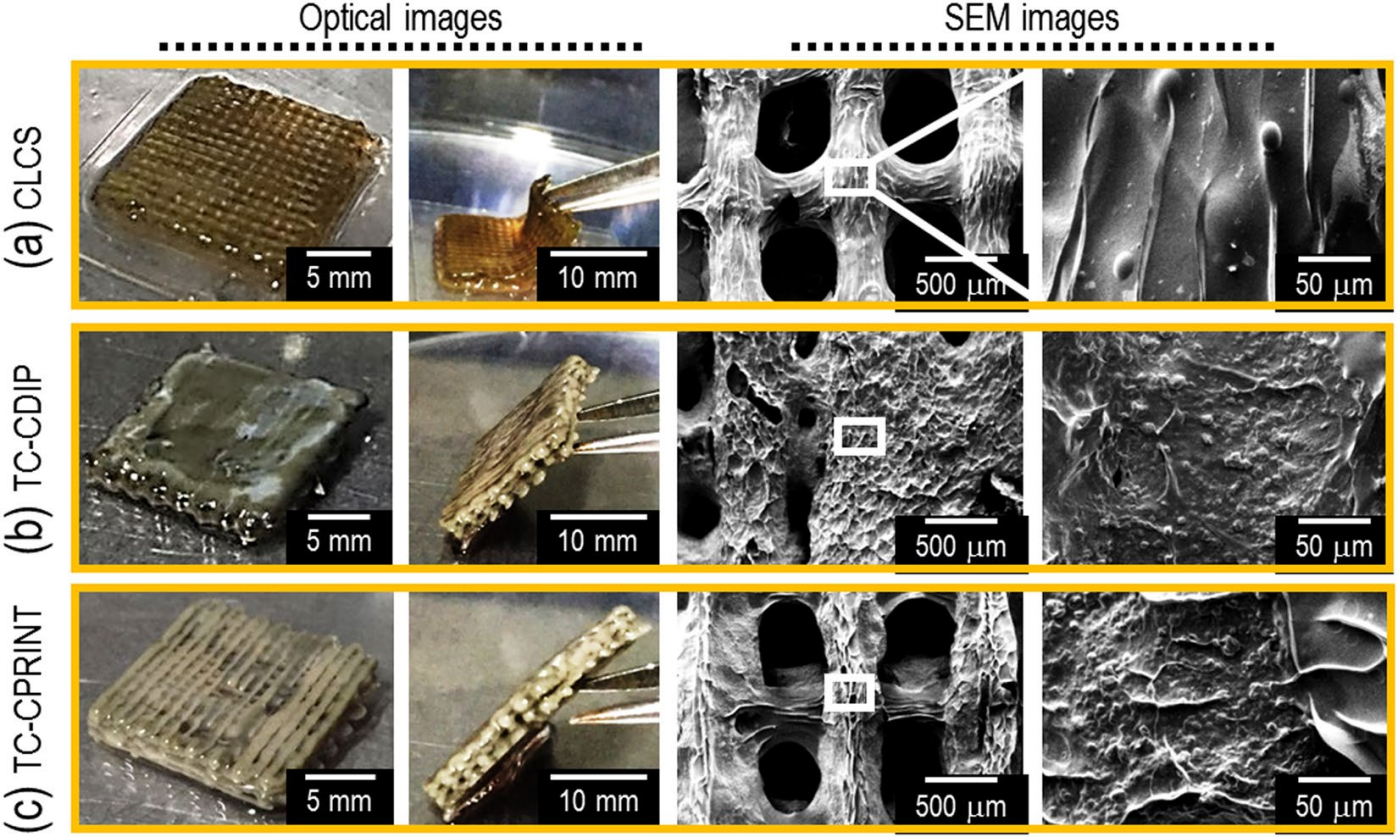
Optical and SEM images of fabricated cell-laden scaffolds. (**a**) Cell-laden collagen scaffold (CLCS), (**b**) TC-CDIP scaffold fabricated by dipping the α-TCP/collagen mesh structure into the cell/collagen mixture solution, and (**c**) TC-CPRINT fabricated using the two-step process: printing of α-TCP/collagen struts and then printing cell-laden collagen onto the α-TCP/collagen struts.

To regenerate bone tissues, the structure of the pores (pore size, porosity, permeability, and tortuosity) of scaffolds is an essential design factor because pore geometry directly influences the flow of nutrients and metabolic activities in the process of cell mineralization^24^. Generally, representative scaffold structure needs to possess high porosity and appropriate pore size (over 100 μm), as well as high pore-connectivity and tortuosity^18^. Under appropriate processing conditions, it is possible to fabricate cell-loaded scaffolds; Fig. 5a shows the optical and scanning electron microscopy (SEM) images of a CLCS scaffold, which was crosslinked with TA (2 wt%). Figure 5b and c show two cell-laden α-TCP/collagen scaffolds; in the TC-CDIP scaffold, the cells were loaded by dipping the mesh structure (α-TCP/collagen) into the cell-collagen solution, while the TC-CPRINT scaffold was fabricated using cell printing on α-TCP/collagen struts. The details for the fabricating conditions, and the composition of the three scaffolds, are described in Table 1. The CLCS and TC-CPRINT scaffolds were porous scaffolds with homogeneous pore size, while TC-CDIP had a non-homogenous pore structure, caused by the blockage of the cell-laden collagen solution during the dipping process.

**Table 1:** Fabricating conditions and composition of CLCS, TC-CD, and TC-CP.

Composition	CLCS	TC-CDIP	TC-CPRINT	
	Cell-laden collagen	α-TCP/collagen struts	α-TCP/collagen struts	Cell-laden collagen
Volume fraction of α-TCP (v/v%)	—	80	80	—
Weight fraction of TA (wt%)	2	1	1	0
Nozzle dia. (μm)	250	250	250	150
Nozzle moving speed (mm s^−1^)	10	10	10	10
Pneumatic pressure (kPa)	230	120	120	180

### Characterization of the α-TCP/collagen scaffold

As controls, we used the CLCS and TC-CDIP and compared the physical and bioactive properties of the controls with the experimental group (TC-CPRINT).

**Figure 6:**
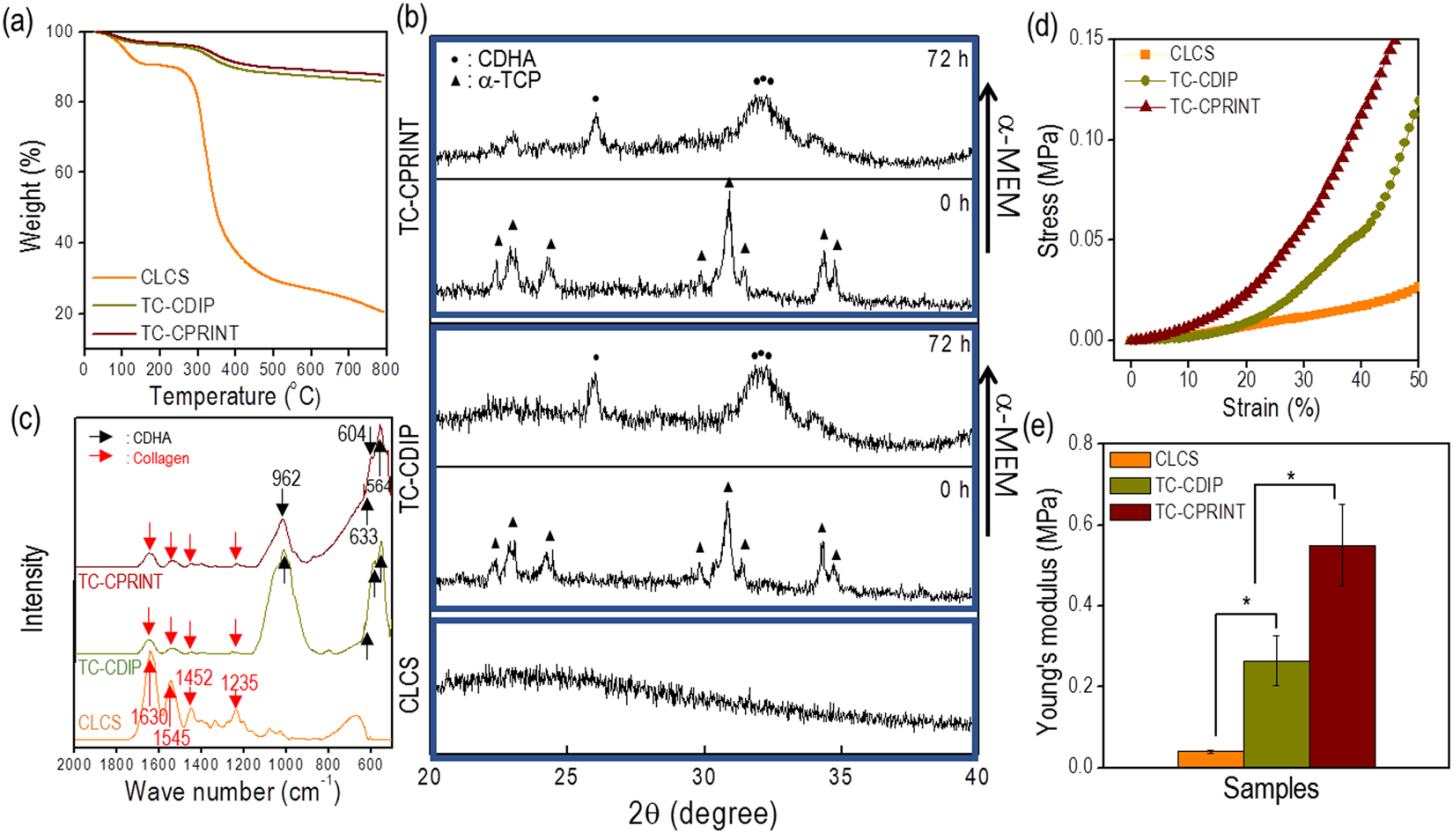
Characterizations of cell-laden scaffolds. (**a**) Thermogravimetric analysis and (**b**) Fourier-transform infrared spectroscopy (FTIR) of CLCS, TC-CDIP, and TC-CPRINT scaffolds. (**c**) The results of X-ray diffraction (XRD) for the TC-CDIP and TC-CPRINT scaffolds before and after the cement reaction, and the result for CLCS. (**d**) Compressive stress-strain curves and (**e**) compressive moduli of the scaffolds. The asterisk indicates significant difference.

Thermogravimetric analysis (TGA) was used to characterize the weight composition of the scaffolds. As shown in Fig. 6a, the results of TGA indicate that collagen was degraded at the temperature range of 370–390 °C and remnant weight was used to determine the percentage of residual α-TCP. The curves show the measurements of remnant α-TCP (69 ± 4.2 v/v% in the TC-CDIP scaffold and 71 ± 1.5 v/v% in the TC-CPRINT scaffold). The remnant amount of α-TCP differed slightly among the scaffolds; however, because two different processes were used (dipping for loading the cell-laden collagen bioink and cell printing of cell-laden collagen solution), the different volume fraction may be negligible. Therefore, the α-TCP volume fraction of the scaffolds was assumed to be similar.

Figure 6b shows the typical fourier-transform infrared (FTIR) spectra of CLCS, TC-CDIP, and TC-CPRINT scaffolds. In the CLCS, the spectra showed an amide-I band at 1630 cm^−1^, amide-II bands at 1543 and 1452 cm^−1^, and amide-III bands at 1235 cm^−1^. A new band appeared at 1545 cm^−1^, caused by the formation of new hydrogen bonds between collagen and tannic acid^25^. Additionally, the spectra of α-TCP/collagen scaffolds demonstrated the typical FTIR spectra for collagen and α-TCP. According to Siddharthan^26^, the characteristic vibration bands of α-TCP were PO_4_^3−^ groups (564, 603, 962, and 1032 cm^−1^) and structural OH^−^ (633 and 3570 cm^−1^); α-TCP and collagen was well constituted in the both scaffolds.

Figure 6c shows the X-ray diffraction (XRD) data for the CLCS and TC-CDIP and TC-CPRINT scaffolds before and after the cement reaction in α-MEM for 72 h. During the analysis of diffraction peaks, the α-TCP/collagen scaffolds showed the representative XRD peaks indicative of the presence of α-TCP, which possesses an orthorhombic crystal structure. However, after immersing the scaffolds in α-MEM for 72 h, the α-TCP component underwent hydrolysis and cementation. Therefore, the crystal peaks of α-TCP were changed to those of calcium-deficient hydroxyapatite (CDHA), which is similar to the inorganic constituents in bone^16^. As shown in the XRD patterns, after the cement reaction, the CDHA peaks were clearly visible in the α-TCP/collagen scaffolds.

Compressive mechanical properties of scaffolds are an important factor affecting bioactivities such as the morphology of cultured cells, differentiation, and endurance with respect to exterior mechanical loading^27^. Figure 6d shows the compressive stress-strain curves of the three scaffolds at the compression rate of 0.5 mm s^−1^ in a wet state. The results show that the elastic modulus of CLCS was 0.04 ± 0.004 MPa, while the moduli of TC-CPRINT and TC-CDIP were 0.55 ± 0.10 MPa and 0.26 ± 0.06 MPa, respectively (Fig. 6e). Because of the high volume fraction of the ceramic component, the compressive modulus of the α-TCP/collagen scaffold was significantly improved compared to that of pure cell-laden collagen scaffold (CLCS). However, because of the pore structure of the scaffolds, the mechanical properties of both α-TCP/collagen scaffolds were low compared with those (0.02~0.52 GPa) of real trabecular bone whose range of density is from 0.09~0.75 g cm^−3^ ^28^. Overcoming low mechanical properties is the next challenge in the fabrication of α-TCP/collagen scaffolds. Currently, α-TCP/collagen scaffolds can function biologically as impermanent mechanical sustainers; however, they can be degraded during clinical applications.

### *In vitro* cellular activities of the cell-printed ceramic scaffold

**Figure 7:**
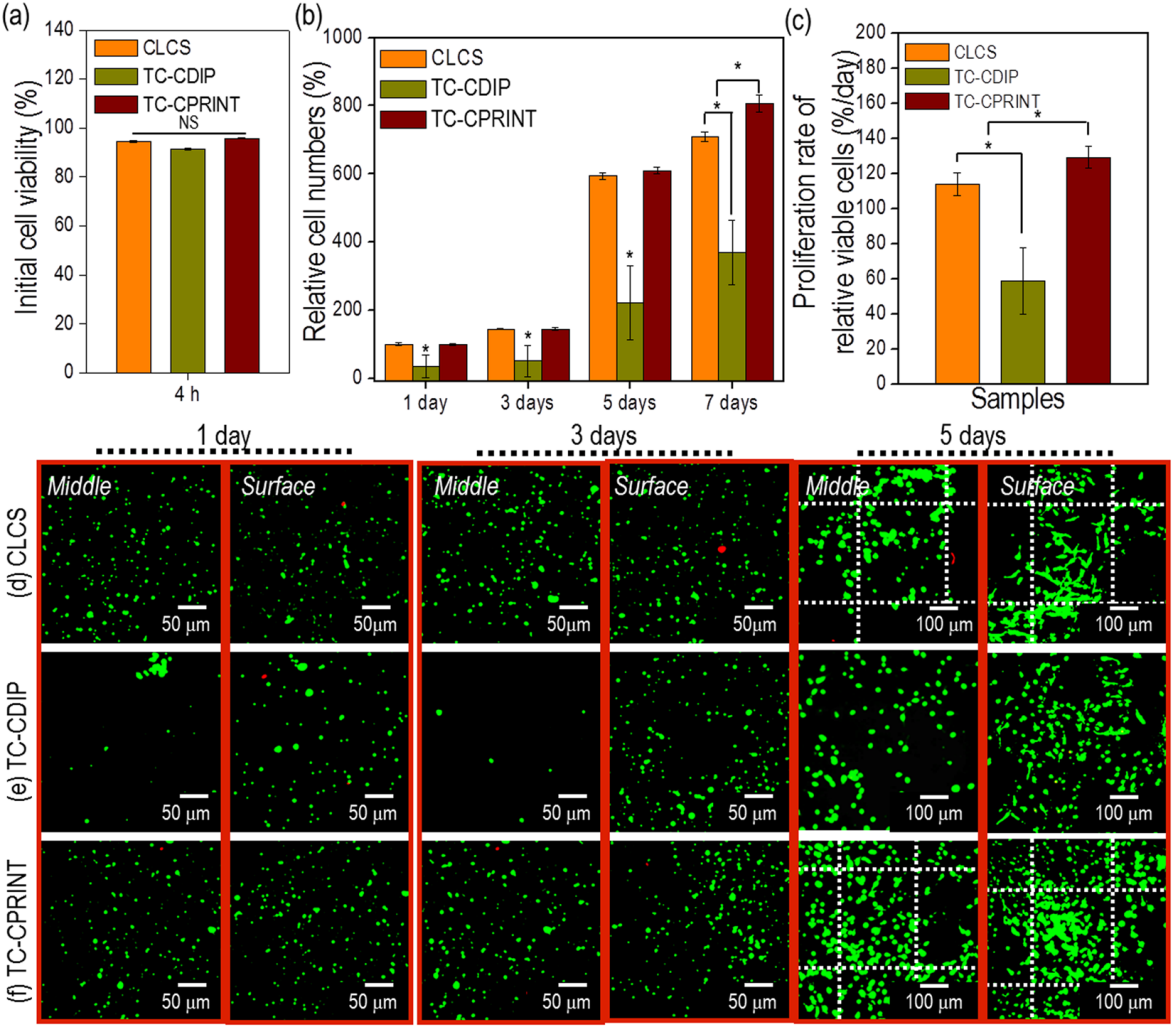
*In vitro* cell-activities and live/dead images of the cell-laden scaffolds. (**a**) Initial cell viability at 4 h, (**b**) relative cell numbers, per the identical area for various cell-culture periods, show cell proliferation; (**c**) the rate of cell proliferation calculated using relative viability cell numbers; and (**d**) live-dead images (of the middle and surface regions) of the scaffolds cultured for various periods. The asterisk and NS indicate significant difference and non-significance, respectively.

*In situ* cell viability after cell printing is an important factor for successful tissue regeneration because it directly influences cell proliferation. After printing cell-laden collagen onto the α-TCP/collagen struts, the viability at 4 h was measured using the live (calcein AM; green)-dead (ethidium homodimer-1; red) staining. Figure 7a shows that cell viability was above 91% for all the scaffolds. This indicates that the cell-printing and -dipping processes were not detrimental to the cells.

The proliferation of viable cells was determined, using the live-dead staining for the CLCS, TC-CDIP, and TC-CPRINT scaffolds, for various culture periods (Fig. 7b). The number of viable cells on the TC-CPRINT and CLCS scaffolds was considerably higher compared with that on the TC-CDIP scaffold. By plotting the cell numbers vs. the culture period, we calculated the proliferation rate using simple linear regression. The cells on the CLCS, TC-CDIP, and TC-CDIP scaffolds showed the proliferation rates (%/day) of approximately 112, 58, and 128, respectively. This indicates that the CLCS and TC-CPRINT scaffolds can provide favorable micro-cellular environmental conditions, promoting cell-cell interactions. This may have occurred because of homogenous cell distribution, which was obtained by printing the cells directly onto the CLCS and TC-CPRINT scaffolds. To assess cell distribution, we compared the live-dead images taken of the surface and middle of the TD-CP and TD-CD scaffolds. Images of live-dead staining in the middle and surface areas show that the CLCS and TD-CP scaffolds had a more homogenous cell distribution compared with that of the TD-CD scaffold (Fig. 7d–f).

**Figure 8:**
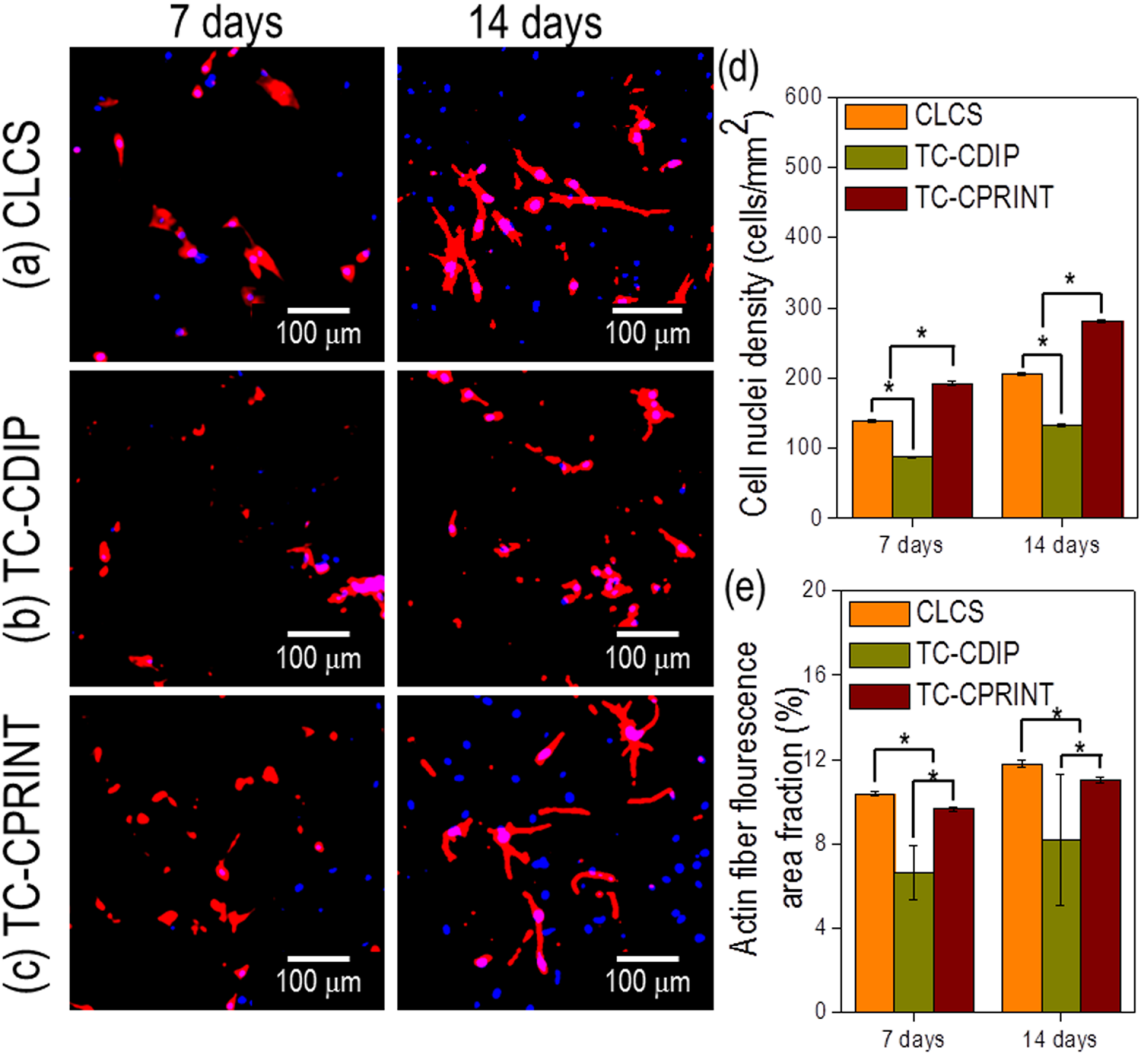
DAPI/phalloidin staining after 7 and 14 days of culture of the scaffolds. DAPI/phalloidin images of (**a**) CLCS, (**b**) TC-CDIP, and (**c**) TC-CPRINT. (**d,e**) Images showing the number of nuclei and levels of F-actin in the areas of interest, visualized using DAPI/phalloidin. The asterisk indicates significant difference.

Figure 8a–c shows the nuclei (blue)/cytoskeleton (red) of the cells cultured on the scaffolds for 7 and 14 days. We measured the number of nuclei per mm^2^ and the area of F-actin on the scaffolds. Similar to the result for the rate of cell proliferation, the number of nuclei and cytoskeletal area of the cells grown on the cell-printed scaffolds (CLCS and TC-CPRINT) were significantly increased compared with that of cells grown on the TC-CDIP scaffold.

**Figure 9:**
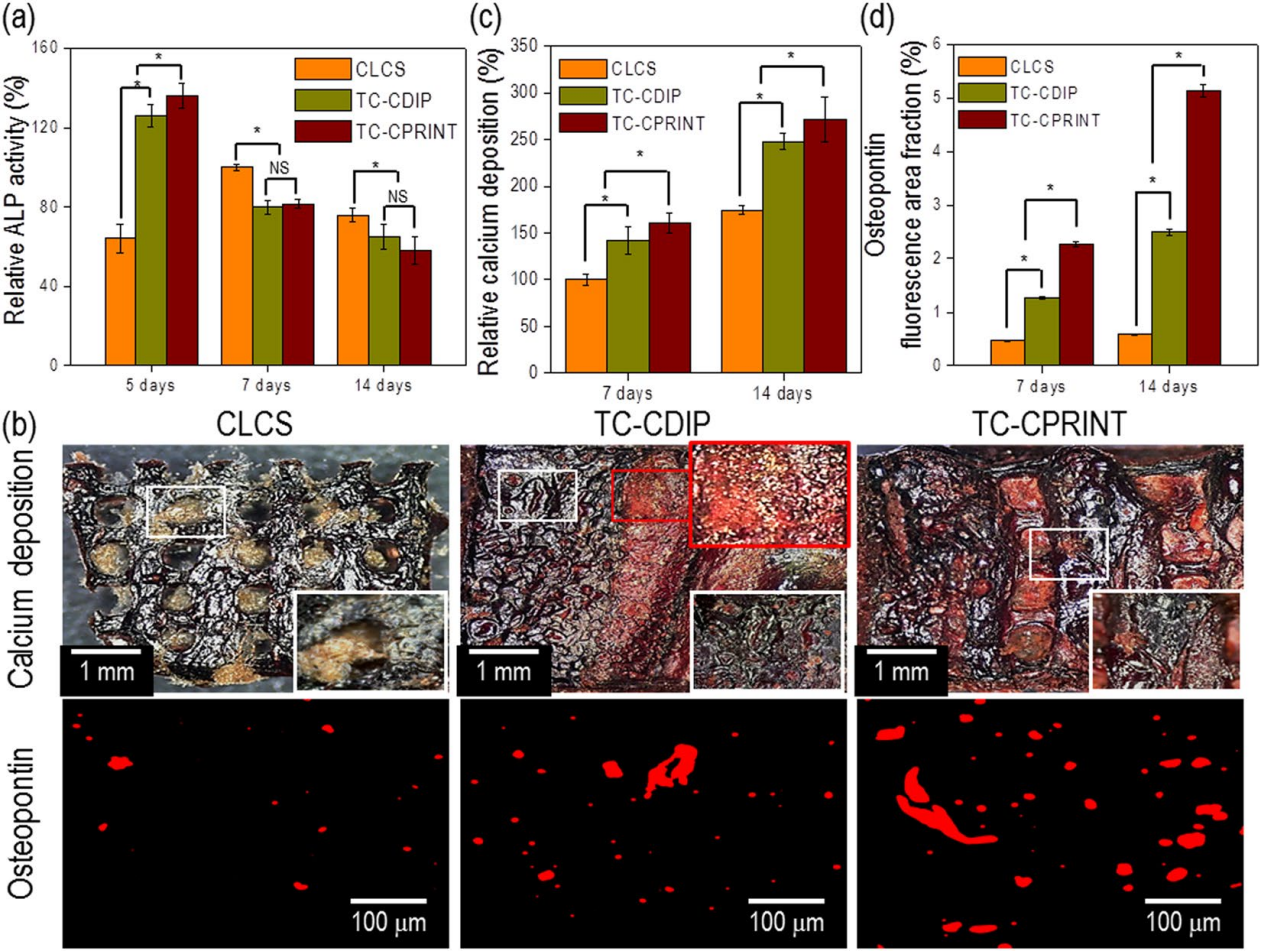
Osteogenic activites of cell-laden scaffolds. (**a**) Relative alkaline phosphatase (ALP) activity. (**b**) Optical images of Alizarin Red S (ARS) and osteopontin (OPN) staining of the scaffolds after cell culture for 14 days. (**c**) Relative calcium deposition and (**d**) relative area of OPN images of scaffolds (n = 5). The asterisk and NS indicate significant difference and non-significance, respectively.

To assess osteogenic differentiation of the scaffolds, we evaluated alkaline phosphatase (ALP) activity, osteopontin (OPN), and calcium deposition using Alizarin Red staining. ALP activity was normalized to total protein content (Table 2); the activity of the scaffolds decreased progressively starting on day 5 of culture, showing a particular decrease on days 7 and 14; this is because the activity of ALP occurs in the early stages of bone differentiation. Among all the scaffolds at 5 days of culture, the TC-CPRINT scaffold showed the highest activity of ALP (Fig. 9a).

**Table 2:** Total protein contents of the scaffolds.

	CLCS (mg)	TC-CDIP (mg)	TC-CPRINT (mg)
7 days	59.6 ± 1.9	69.5 ± 2.3	78.7 ± 3.0
14 days	85.9 ± 3.3	95.4 ± 3.2	121.7 ± 3.9

The optical images of stained scaffolds (calcium deposition and OPN) were evaluated after 14 days of cell culture (Fig. 9b). A significant level of bright red color and high calcium deposition were observed in the TC-CPRINT scaffold compared with those in the CLCS and TC-CDIP scaffolds.

Calcium dissolution assay was used to determine calcium deposition in the scaffolds; the value was normalized to the total protein content of each scaffold, and measured fluorescence area of OPN for the scaffolds was described in Fig. 9c and d. The results of *in vitro* bioactivity assay showed synergism between the homogenously printed cells and the calcium and phosphorus ions released from the CDHA scaffold; this can directly affect osteogenic differentiation in the TC-CPRINT scaffold, indicating that the TC-CPRINT scaffold may be an effective bioactive platform for the regeneration of bone tissue.

## Conclusion

Here, we developed an innovative ceramic-based scaffold, which consisted of α-TCP/collagen and cell-printed collagen, and was fabricated using 3D printing combined with a cell-printing process. We found an optimal processing range for the parameters used in the fabrication, including the weight fraction of tannic acid and α-TCP, nozzle size, and pneumatic pressure. Using these settings, we effectively manufactured a cell-laden α-TCP/collagen scaffold. A cell-laden collagen scaffold, and an α-TCP/collagen scaffold dipped into cell-laden collagen solution, were used as controls. The physical properties and bioactivities of the scaffolds indicated that the cell-printed α-TCP/collagen scaffold demonstrated significantly higher mechanical properties and cellular activities compared with those of the pure cell-laden collagen scaffold and α-TCP/collagen scaffold dipped into cell-laden collagen solution. Our results indicate that the cell-printed α-TCP/collagen scaffold may serve as potential biomaterial for the regeneration of bone tissue.

## Experimental

### Cells and materials

α-TCP was kindly provided by Dr. H-S Yun (Powder and Ceramics Division, Korea Institute of Materials Science, South Korea). Type I collagen, derived from porcine tendon (Matrixen-PSP; SKBioland, South Korea) was used as bioink. To create a neutral collagen solution, 10 × enriched DMEM solution was mixed with a collagen solution at a volume ratio of 1:1^21^. The neutralized collagen (4 wt%) was mixed with MC3T3-E1 cells (ATCC, Manassas, VA, USA) at the density of 1 × 10^6^ cells mL^−1^. To cross-link the collagen, with and without the cells, the collagen solution was mixed with various weight fractions of tannic acid ([TA] Sigma-Aldrich, St. Louis, MO, USA).

### Fabricating conditions for cell-laden collagen scaffold and cell-loaded α-TCP/collagen scaffolds

The CLCS scaffold was fabricated using a dispensing system (DTR2–2210 T; Dongbu Robot, Bucheon, South Korea) with a nozzle (inner diameter = 200 μm) and a three-axis printing system (DTR3-2210 T-SG, DASA Robot, Seoul, South Korea) (Fig. 1a). The applied pneumatic pressure in the barrel/nozzle was 230 kPa. The cell-laden collagen scaffold was immersed in α-MEM medium and washed in phosphate buffered saline (PBS). The moving speed of the printing nozzle in the printing system was set at 10 mm·s^−1^.

To fabricate a TC-CDIP scaffold, a 3D α-TCP/collagen mesh structure was dipped into a cell-laden collagen solution (4 wt% collagen and MC3T3-E1 cells at the density of 1 × 10^6^ cells mL^−1^) (Fig. 1b). Also, after printing the α-TCP/collagen struts shown in Fig. 1c, the cell-laden collagen solution was printed onto the struts. These steps were repeated several times to achieve a TC-CPRINT scaffold. Finally, the TC-DIP and TC-CPRINT were rinsed in α-MEM medium and PBS. After rinsing, the scaffolds were immersed in α-MEM solution for 72 h at 37 °C to conduct hydrolysis of α-TCP.

### Characterization of the bioink and scaffolds

Cell-laden collagen (which included MC3T3-E1 cells at the density of 1 × 10^6^ mL^−1^), crosslinked using various concentrations of tannic acid, was used to evaluate the rheological properties such as storage modulus (G′) and complex viscosity (n*). The properties were assessed using a rotational rheometer (Bohlin Gemini HR Nano; Malvern Instruments, Surrey, UK) with cone-and-plate geometry (40 mm diameter, 4° cone angle, 150-μm gap). A frequency sweep was conducted within the linear viscoelastic region at 30 °C with 1% strain.

The surface morphology of the scaffolds was characterized by scanning electron microscopy (SEM) (SNE-3000M, SEC Inc., Seoul, South Korea) using an optical microscope (Model BX FM-32; Olympus, Tokyo, Japan) connected to a digital camera. Using the optical and SEM images, the pore size was defined as the distance between the struts.

Thermogravimetric analysis (TGA) was conducted under nitrogen atmosphere using TGA-2050 (TA-Instruments, New Castle, DE, USA). A typical sample mass of 10 mg was heated from 30 °C to 900 °C at a ramp rate of 20 °C min^−1^.

Fourier-transform infrared (FTIR) spectroscopy (model 6700; Nicolet, West Point, PA, USA) was conducted to assess the properties of the materials used during the fabrication of the bioinks. Infrared (IR) spectra were used to perform the average of 30 scans in the range of 500−2000 cm^−1^ at the resolution of 10 cm^−1^.

Wide-angle X-ray diffraction (Siemens D500 WAXD, Munich, Germany), with CuKα radiation under the beam conditions of 40 kV and 20 mA, with collection of the spectrum at 2θ = 20~40° and a step size of 0.1°, was performed to obtain the crystal peaks of α-TCP and calcium-deficient hydroxyapatite (CDHA).

The mechanical properties of the scaffolds were assessed in compressive mode and wet state. The test was conducted using a universal testing instrument (Top-tech 2000; Chemilab, Seoul, South Korea) at 30 °C. The compressive stress-strain curves, for the cell-laden mesh structure, were recorded at a compression rate of 0.2 mm·s^−1^ (diameter = 6 mm; thickness = 2.3 mm). All values are expressed as means ± SD (*n* = 5).

### *In vitro* cell culture

The cell-loaded scaffolds were cultured in 6-well culture plates using minimum essential medium eagle-alpha (α-MEM) supplemented with 10% fetal bovine serum (FBS) and 1% penicillin-streptomycin (Thermo Fisher Scientific, USA). The scaffolds were preserved in α-MEM with 5% CO_2_ at 37 °C, and the medium was changed every 2 days. T induce differentiation, the cells were cultured in α-MEM, containing 50 μg mL^−1^ vitamin C and 10 mM β-glycerophosphate, after 7 days of culture.

### Fluorescence Imaging

The scaffolds were exposed to 0.15 mM calcein AM and 2 mM ethidium homodimer-1 for 45 min in an incubator to observe live and dead cells. The stained specimens were visualized under a confocal microscope (LSM 700, Carl Zeiss, Germany). The stained images were captured, the green and red colors indicating live and dead cells, respectively. ImageJ software (National Institutes of Health, Bethesda, MD) was used to count the live cells.

After 7 and 14 days of cell culture, the scaffolds were subjected to diamidino-2-phenylindole (DAPI) fluorescence staining to detect the cell nuclei. Phalloidin (Invitrogen, Carlsbad, CA) staining was performed to visualize the actin cytoskeletons of proliferated cells. Images were obtained using a confocal microscope.

### Total protein content

The total protein content was measured by the BCA protein assay (Pierce kit; Thermo Scientific). The scaffolds were washed with PBS and lysed with 1 mL of Triton X-100 (0.1%). An aliquot of the lysate (25 μL) was added to 200 μL of BCA working reagent, and the mixture was incubated for 30 min at 37 °C. Protein concentration was determined using absorbance at 562 nm; the absorbance was measured using an enzyme-linked immunosorbent assay reader and converted to total protein concentration using a standard curve.

### Osteogenic activities

ALP was assayed by measuring the release of p-nitrophenol (pNP) from p-nitrophenyl phosphate (pNPP). The cultured scaffolds were rinsed gently with PBS and incubated for 10 min in Tris buffer (10 mM, pH 7.5) containing 0.1% Triton X-100 surfactant. Then, 100 μL lysates were added to the wells of 96-well tissue culture plates containing 100 mL of pNPP solution prepared using an ALP kit (procedure no. ALP-10; Sigma-Aldrich). In the presence of ALP, pNPP is converted to pNP and inorganic phosphate. ALP activity was determined from the absorbance at 405 nm using a microplate reader (Spectra III; SLT Lab Instruments, Salzburg, Austria). The levels of ALP activity were normalized by the total protein content in the media of the respective wells. All data values are defined as means ± SD (n = 5).

Calcium mineralization was evaluated by staining MC3T3-E1 cells with Alizarin Red S (ARS) in 24-well culture plates. The cultured sample was rinsed three times with PBS, followed by fixation using 70% (v/v) cold ethanol at 4 °C for 1 h; the sample was then air-dried. The sample was stained with 40 mM Alizarin Red S (pH 4.2) for 1 h and rinsed three times with purified water. Lastly, the sample was destained with 10% cetylpyridinium chloride in 10 mM sodium phosphate buffer (pH 7.0) for 15 min. The stained sample was imaged using an optical microscope, and the absorption at 562 nm was measured using a Spectra III UV microplate reader. The measured levels of calcium deposition were normalized by the total protein content in the media of the respective wells. All data values are defined as means ± SD (n = 5).

Osteopontin (OPN) staining was measured using the protocol proposed by Park *et al*.^22^. The scaffolds were fixed with 3.8% (w/v) formaldehyde (Sigma aldrich, St. Louis, MO, USA) in phosphate-buffered saline (PBS) for 20 min at room temperature. For permeabilization of the cells, 0.1% (v/v) Triton-X 100 (Sigma aldrich, St. Louis, MO, USA) in PBS was added for 5 min, and the cells were washed three times with PBS. To block nonspecific binding of the antibodies, the cells were incubated with 2% (v/v) normal goat serum (Sigma aldrich, St. Louis, MO, USA) in PBS for 30 min at room temperature and washed three times with PBS. The scaffolds were incubated with primary antibodies (anti-osteopontin antibodies, Abcam, Cambridge, UK) at 4 °C overnight. After incubation with primary antibodies, the cells were washed three times with PBS. Alexa Fluor 168-conjugated goat anti-mouse secondary antibodies (Invitrogen, Carlsbad, CA, USA) were added to the cells, and the cells were incubated for 1 h at room temperature. The fluorescence signals were observed under a confocal microscopy.

### Statistical analyses

All data are presented as means ± standard deviation. Statistical analyses were performed using SPSS software (version 20.0; SPSS, Inc., Chicago, IL) and included single-factor analyses of variance (ANOVA). In all the analyses, *P* values < 0.05 were considered statistically significant. “NS” indicates no statistically significant difference.
